# Popliteal Venous Aneurysm with Recurrent Pulmonary Embolism Treated by Ligation without Venous Reconstruction: A Case Report

**DOI:** 10.70352/scrj.cr.26-0519

**Published:** 2026-11-05

**Authors:** Masashi Bungo, Ken-ichi Watanabe, Yoshio Teshima, Yuji Sakashita, Hisashi Uemura, Mitsuhiro Yamamura, Taichi Sakaguchi

**Affiliations:** Department of Cardiovascular Surgery, Hyogo Medical University, Nishinomiya, Hyogo, Japan

**Keywords:** popliteal venous aneurysm, pulmonary embolism, syncope, venous thrombosis, venous ligation

## Abstract

**INTRODUCTION:**

Popliteal venous aneurysm (PVA) is rare but may lead to pulmonary embolism (PE).

**CASE PRESENTATION:**

A 73-year-old man presented with recurrent, transient loss of consciousness. Contrast-enhanced CT demonstrated bilateral PE and a saccular aneurysm of the right popliteal vein. Anticoagulation therapy improved his respiratory status, but surgical treatment was performed in view of the risk of recurrent embolism. The postoperative course was uneventful.

**CONCLUSIONS:**

At the 1-year follow-up, the patient remained free of clinically apparent lower-leg edema, and venous duplex ultrasonography demonstrated no deep vein thrombosis and the development of collateral venous channels. Although ligation is not a standard procedure for PVA, it may be an effective option in patients in whom reconstruction is difficult or in those at high operative risk.

## Abbreviations

PE
: pulmonary embolism
PVA
: popliteal venous aneurysm
TALV
: tangential aneurysmectomy with lateral venorrhaphy
VTE
: venous thromboembolism

## INTRODUCTION

PVA is a relatively uncommon disease defined as a localized venous dilatation without tortuosity or valvular insufficiency, which distinguishes it from lower-extremity varicose veins. PVA is clinically significant because it can be an important source of deep vein thrombosis and PE. When PVA is accompanied by thrombus, anticoagulation alone may be insufficient to prevent recurrent PE, and surgical treatment is recommended for symptomatic cases or those with high-risk morphology.

Here, we report a case of acute PE with recurrent transient loss of consciousness, in which PVA was considered the embolic source. The patient was successfully treated with aneurysmotomy, thrombectomy, and proximal and distal ligation without venous reconstruction. The educational value of this case lies in the decision-making process for selecting ligation without reconstruction when venous reconstruction was considered technically unfavorable, as well as in the recurrence of PE after a 5-year delay in definitive treatment following the initial diagnosis of PVA.

## CASE PRESENTATION

A 73-year-old man was emergently transferred to the hospital after a syncopal episode and dyspnea. He had experienced a similar syncopal event 5 years earlier and had been admitted to another hospital, where he was diagnosed with PE. At that time, a PVA was identified, and surgical treatment was planned. However, the patient discontinued outpatient follow-up before undergoing definitive treatment and was subsequently lost to follow-up. Details of the anticoagulation therapy administered at the previous hospital were unavailable. On arrival, the patient had labored breathing; his respiratory rate was 18 breaths/min, oxygen saturation was 95% on 10 L/min via face mask, and blood pressure was 102/73 mmHg. Contrast-enhanced CT on arrival revealed thrombi extending from the central pulmonary arteries to the peripheral branches bilaterally (**Fig. 1A**). The right popliteal vein was dilated to approximately 32 mm, and mural thrombus was identified within the aneurysm (**Fig. 1B** and **1C**). Thrombophilia screening, including protein C activity, protein S activity, antithrombin activity, lupus anticoagulant, anticardiolipin antibodies, and anti-β2-glycoprotein I antibodies, revealed no abnormalities suggestive of an underlying thrombophilic disorder. Electrocardiography on admission showed normal sinus rhythm. Transthoracic echocardiography demonstrated no right ventricular enlargement, and the tricuspid regurgitation pressure gradient was 19 mmHg, with no apparent echocardiographic evidence of significant right heart strain. Head CT revealed no intracranial lesions that could account for the transient loss of consciousness. The patient was diagnosed with PE originating from a right PVA, and oral apixaban at 20 mg/day was initiated. Dyspnea improved promptly. On hospital day 7, lower-extremity venous ultrasonography confirmed reduced thrombus within the PVA, and the apixaban dose was reduced to 10 mg/day in accordance with the standard dosing regimen for VTE. On hospital day 13, repeat contrast-enhanced CT demonstrated residual thrombi in the peripheral pulmonary arteries and within the PVA. As the lower-extremity venous thrombus was confined to the distal veins without proximal extension and therapeutic anticoagulation could be continued, an inferior vena cava filter was not placed. After stabilization of the acute PE with anticoagulation, the patient was discharged and subsequently readmitted 27 days later for elective surgical treatment of the PVA to prevent recurrent PE. Apixaban was discontinued on the day before surgery. Surgery was performed with the patient in the prone position. A skin incision of approximately 5 cm was made over the posterior popliteal fossa, and a dark-red saccular PVA protruding posteriorly was exposed. The aneurysm was circumferentially dissected, and the popliteal vein was controlled proximally and distally. After ligation of the proximal normal popliteal vein, the aneurysm sac was opened. A large amount of mobile red thrombus was found within the aneurysm. The thrombi were removed as completely as possible, and the proximal outflow vessel was also ligated (**Supplementary Video 1**). Venous reconstruction was not performed, and the procedure was completed. Postoperatively, the patient had no swelling of the right lower extremity and showed no evidence of recurrent thrombosis; the postoperative course was favorable (**Fig. 2**). Apixaban at 10 mg/day was resumed on POD 1, and the patient was discharged on POD 5. Anticoagulation was continued for 6 months after surgery. Because the PVA, which was considered the source of recurrent PE, had been surgically eliminated and follow-up lower-extremity venous ultrasonography showed no residual or recurrent deep vein thrombosis, the risk of recurrent VTE was reassessed, and anticoagulation was discontinued after 6 months.

**Fig. 1:**
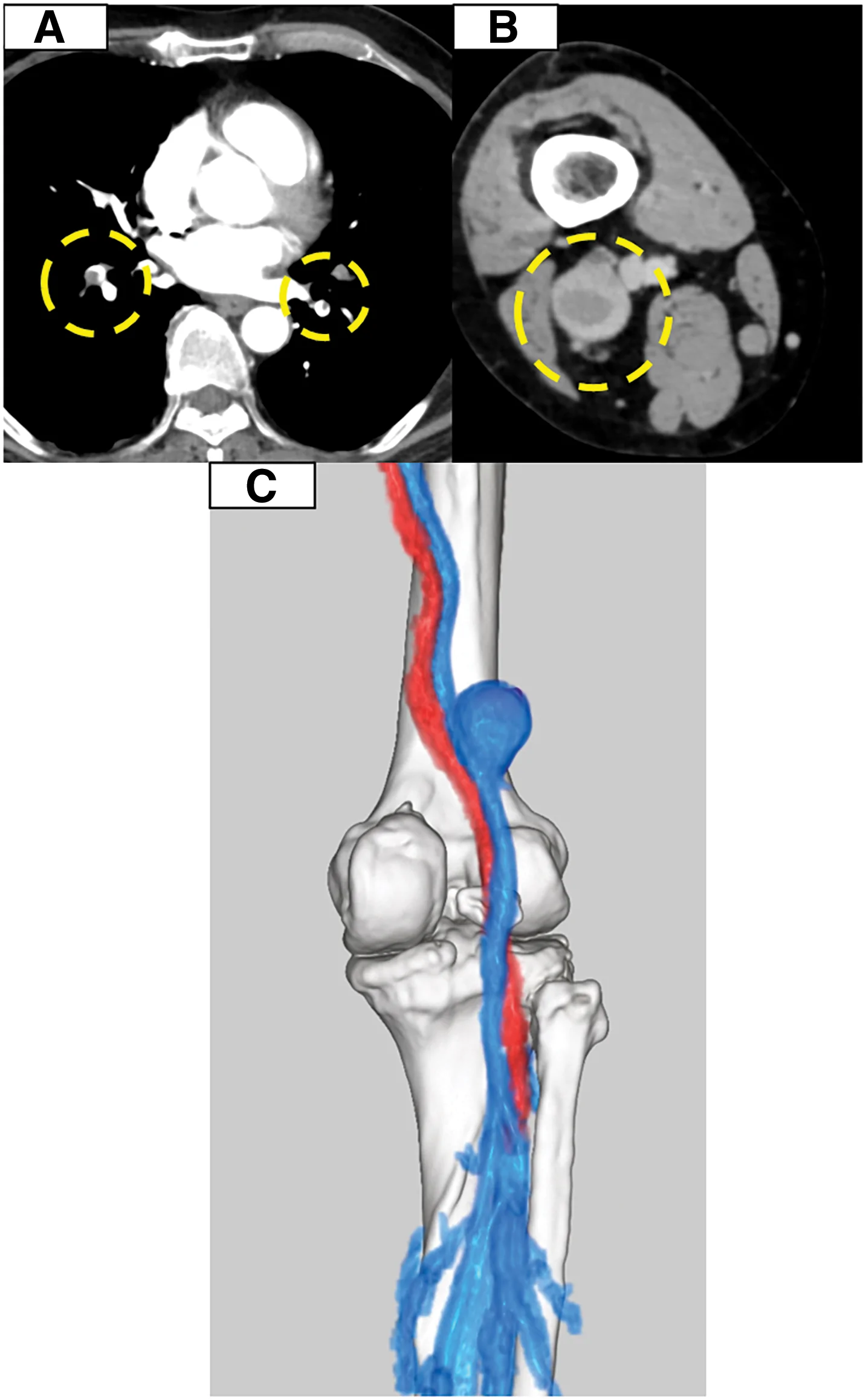
Preoperative imaging findings of PE and PVA. (**A**) Pulmonary arterial thrombi involving the bilateral pulmonary arteries. (**B**) Contrast-enhanced CT and (**C**) 3D reconstruction demonstrating a saccular aneurysm of the popliteal vein with mural thrombus. PE, pulmonary embolism; PVA, popliteal venous aneurysm

**Fig. 2:**
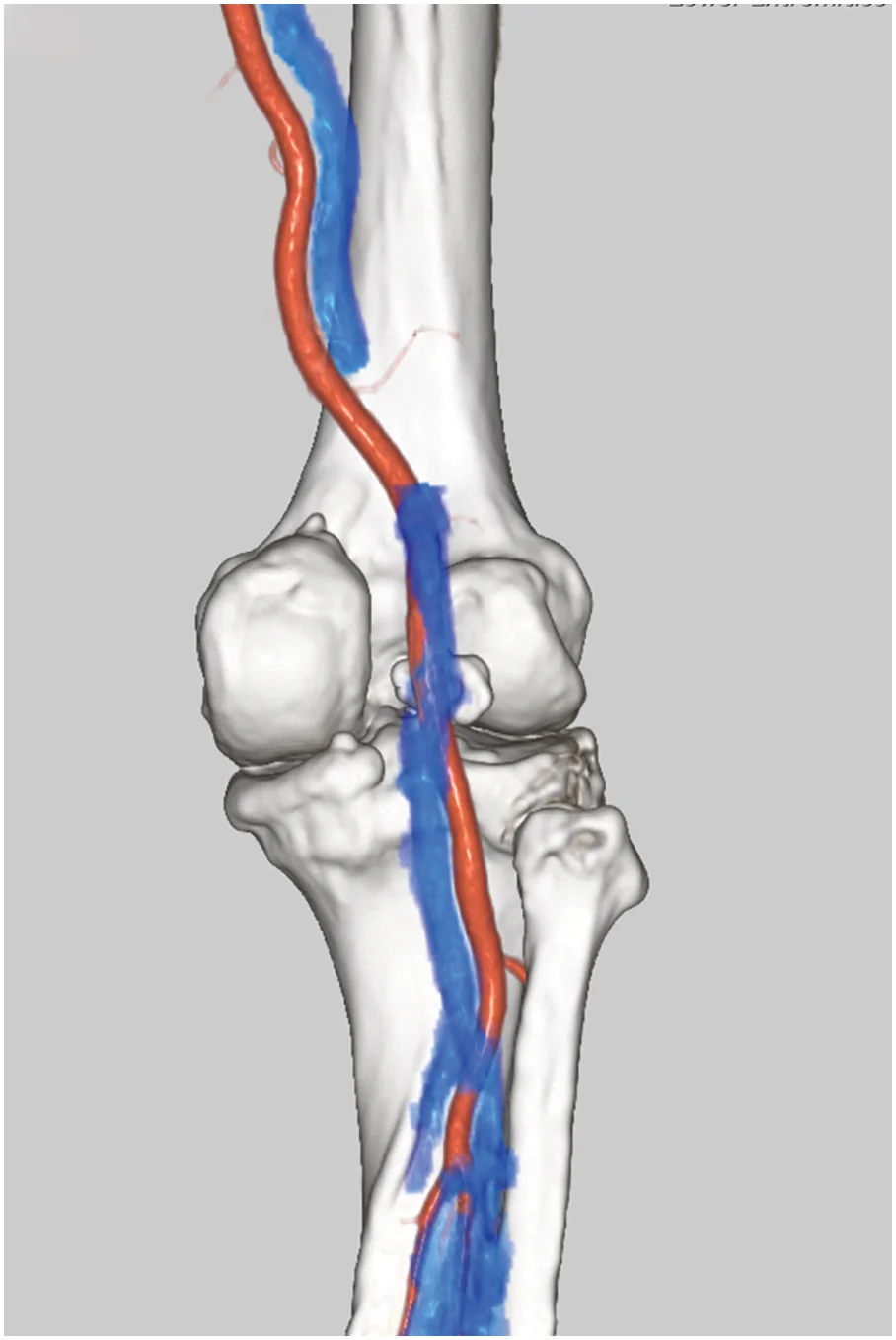
Postoperative venous drainage after popliteal vein ligation. Postoperative 3D CT performed on POD 5 demonstrated preserved venous drainage of the right lower extremity without marked venous congestion compared with that of the contralateral limb.

At the 1-year follow-up, the patient remained free of clinically apparent lower-leg edema during daily activities. Venous duplex ultrasonography demonstrated no deep vein thrombosis and showed the development of collateral venous channels proximal to the ligated popliteal vein (**Fig. 3**).

**Fig. 3:**
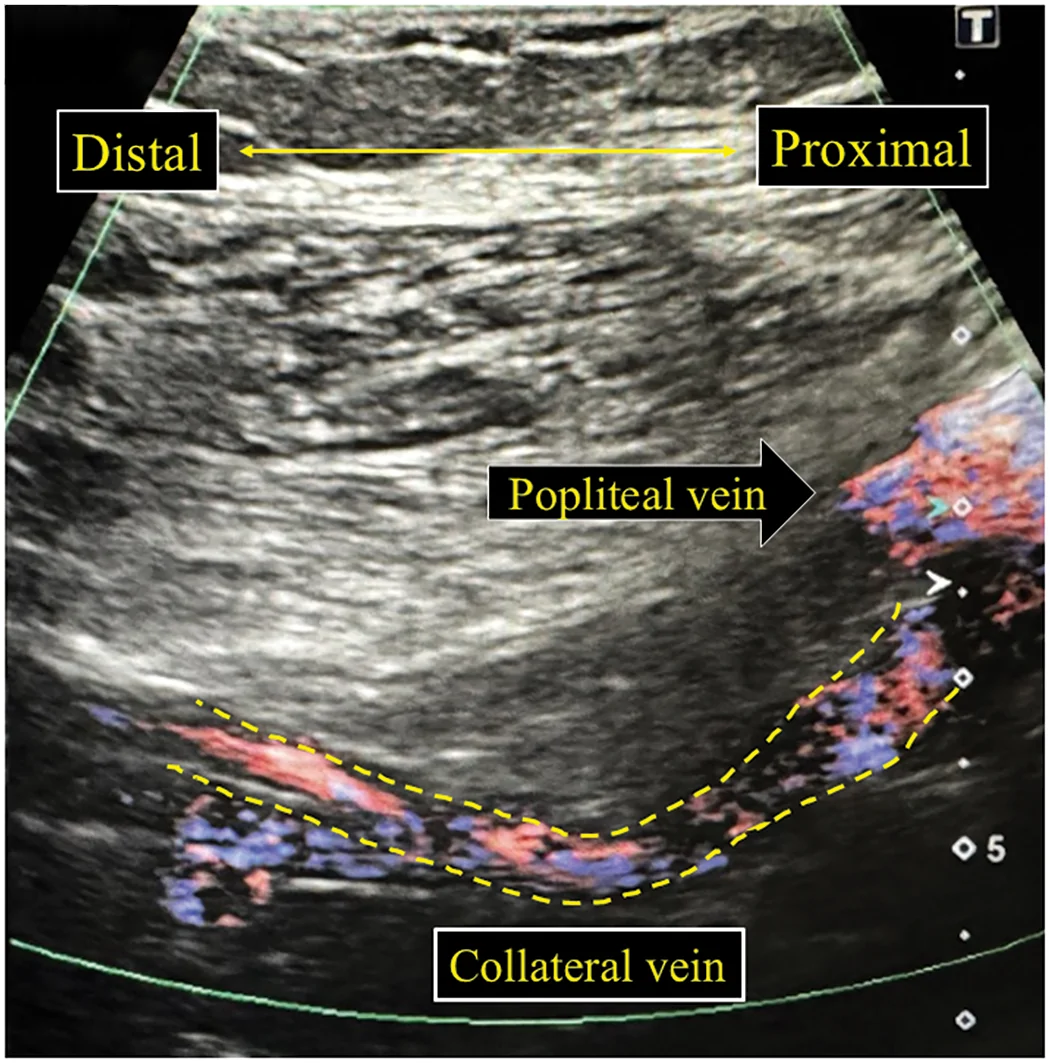
Collateral venous development after popliteal vein ligation. Venous duplex ultrasonography at 1 year postoperatively showed no evidence of deep vein thrombosis and demonstrated the development of collateral venous channels proximal to the ligated popliteal vein.

## DISCUSSION

PVA is a localized dilatation of the popliteal vein without tortuous elongation, and thus differs from lower-extremity varicose veins. The reported prevalence is low (0.1%–0.2%).^1)^ A universally accepted definition has not been established. Maleti et al. proposed diagnostic criteria defining PVA as dilatation of the popliteal vein to 3 times the normal diameter and ≥20 mm in size.^2)^ In the present case, the aneurysm measured 32 mm in diameter and exceeded 3 times the diameter of the adjacent normal popliteal vein, fulfilling Maleti’s criteria.

Syncope in acute PE may result from transient hemodynamic compromise caused by an abrupt increase in pulmonary vascular resistance, leading to reduced cardiac output and cerebral hypoperfusion; a vasovagal reflex may contribute.^3)^ In the present case, mobile thrombi were identified within the PVA, suggesting that recurrent embolization may have caused transient hemodynamic compromise and loss of consciousness. Although CT demonstrated bilateral pulmonary arterial thrombi, transthoracic echocardiography performed on admission showed no right ventricular enlargement, and the tricuspid regurgitation pressure gradient was 19 mmHg. Hemodynamic abnormalities associated with PE-related syncope may resolve rapidly^4)^; therefore, the transient hemodynamic disturbance responsible for the syncope may have resolved by the time of echocardiographic assessment. No arrhythmia was documented on electrocardiography at admission or before surgery, and head CT revealed no intracranial lesions that could account for the transient loss of consciousness. Nevertheless, prolonged electrocardiographic monitoring specifically to evaluate syncope was not performed; therefore, an arrhythmic cause could not be completely excluded. Thus, although PE was considered the most likely cause of the recurrent episodes of transient loss of consciousness, a definitive causal relationship could not be established.

Regarding treatment, anticoagulation alone is generally considered insufficient when symptomatic PE is present, or when a thrombus is identified within a PVA. Therefore, patients with PVA complicated by symptomatic PE are generally considered candidates for surgical treatment.^5)^ No consensus exists on surgical indications for asymptomatic PVA. Patel et al. reported 40 PVA cases from 11 institutions and demonstrated that the mean aneurysm size was larger among patients with deep vein thrombosis or PE than among those without VTE (3.8 cm vs. 2.5 cm; *P* = 0.003).^6)^ Patel et al. also found that saccular morphology was more strongly associated with VTE (30% vs. 9%; *P* = 0.046), and that fusiform aneurysms with thrombus occupying ≥25% of the lumen were likewise associated with VTE (45% vs. 3%).^6)^ These findings suggest that aneurysm size, saccular morphology, and substantial thrombus burden may warrant surgical intervention even in asymptomatic patients.

The primary surgical goal is to remove the aneurysm while maintaining venous return in the affected limb. For saccular aneurysms, the most commonly performed procedure is TALV. When TALV cannot be performed adequately, or in fusiform aneurysms, aneurysm resection with preservation of venous continuity is recommended because residual diseased venous wall may continue to dilate.^7)^ However, reconstructive procedures in PVA have been associated with thrombosis, recurrence, nerve injury, and technical challenges attributable to the limited operative field in the popliteal fossa.

Deep venous ligation has long been reported to be tolerated because venous return can be compensated for by collateral circulation.^8–10)^ Masuda et al. reported that clinically favorable limb function was maintained in most patients during long-term follow-up exceeding 13 years after femoral vein ligation.^10)^ Emmerich et al. reported femoral vein ligation for PVA with recurrent PE and observed no recurrence of PE during >6 years of follow-up, with only mild leg edema and preserved functional status.^11)^ Moreover, studies of traumatic popliteal venous injury suggest that venous ligation does not necessarily worsen short-term outcomes, supporting the limb’s tolerance to the interruption of venous outflow.^12,13)^

Reported outcomes of reconstructive and non-reconstructive surgical strategies are summarized in **Table 1.**^11,14–16)^ Noppeney et al.^14)^ reported favorable long-term outcomes in 29 surgically treated patients, with no postoperative deep venous thrombosis or venous occlusion, although 1 patient developed recurrent PVA after patchplasty. Johnstone et al.^15)^ reported no early complications after aneurysmectomy with lateral venorrhaphy, whereas 2 of 3 patients treated with interposition vein grafting developed early complications. In the systematic review by Borghese et al.,^16)^ ligation was performed in only 6 of 119 surgically treated patients. Although no postoperative PE or 30-day mortality was reported, the outcomes were not stratified by surgical procedure. Therefore, the available evidence does not permit a direct comparison between ligation and reconstruction or establish their equivalence. Ligation should consequently be considered only in carefully selected patients when reconstruction is technically unfavorable and adequate alternative venous drainage has been confirmed.

**Table 1:** Reported outcomes of reconstructive and non-reconstructive surgical strategies for PVAs

Study	Design/cohort	Procedure	Follow-up	Main reported outcomes
Reconstruction				
Noppeney et al. 2019^14)^	Retrospective: 39 patients, 29 underwent surgery*	Direct suture, 23; patchplasty, 1; resection with aneurysmorrhaphy, 3; end-to-end anastomosis, 2	57.9 ± 12.5 months (mean ± SE)	No postoperative DVT/occlusion; PVA recurrence 1/29 after patchplasty (reoperated at 10.5 years); CVI 2; hematoma evacuation 2
Johnstone et al. 2015^15)^	Retrospective: 8 surgical patients	Lateral venorrhaphy, 5; interposition vein graft, 3	26 months	No operative/30-day death; 2 early complications, both after grafting; primary patency 87.5%; secondary patency 100%; no recurrence
Non-reconstructive				
Emmerich et al 2019^11)^	Case report: 1 patient	Femoral vein ligation below the GSV confluence	>6 years	No recurrent PE; normal daily activity; mild ipsilateral leg edema
Present case	Case report: 1 patient	Aneurysmotomy, thrombectomy, and proximal/distal ligation	1 year	No clinically apparent leg edema; no DVT; collateral venous channels developed
Unstratified				
Borghese et al. 2025^16)^	Systematic review: 9 studies; 173 patients; 119 underwent surgery	Vein-preserving/reconstructive, 113; ligation, 6 Outcomes not stratified by procedure	35 months (range 1–262)	Postoperative complications 15/119; no postoperative PE or 30-day mortality; reconstruction thrombosis 1. Pooled outcomes cannot be attributed to either procedure group.

*Noppeney: The 29 surgical patients included 2 with isolated fibular vein aneurysms. A recurrence of 1/29 refers to the entire surgical cohort.

No study directly compared ligation with reconstruction. Because pooled outcomes were not stratified by procedure, relative safety and efficacy cannot be determined.

CVI, chronic venous insufficiency; DVT, deep vein thrombosis; GSV, great saphenous vein; PE, pulmonary embolism; PVAs, popliteal venous aneurysms; SE, standard error

Recurrent transient impairment of consciousness associated with PE secondary to PVA is not unique to the present case. Kawatani et al.^17)^ reported a 75-year-old man with recurrent presyncope caused by PE secondary to a 20-mm PVA; an inferior vena cava filter was placed, followed by TALV to preserve venous continuity. Norimatsu and Aramoto^18)^ reported 3 patients with PVA presenting with severe or recurrent PE, all of whom underwent surgical reconstruction with preservation of venous continuity. In contrast, the present case was characterized by complete loss of consciousness rather than presyncope and by recurrence of PE after a prolonged delay in definitive treatment. Notably, the PVA had already been identified, and surgical treatment had been planned during the initial episode of PE 5 years earlier; however, the patient was lost to follow-up before undergoing definitive treatment. Furthermore, unlike the reconstructive procedures used in previous reports, we performed proximal and distal ligation without venous reconstruction because the large wall defect, the poor quality of the remaining venous wall, and the limited operative exposure made safe and durable reconstruction technically unfavorable. At the 1-year follow-up after ligation, the patient remained free of clinically apparent lower-leg edema, and venous duplex ultrasonography demonstrated no deep vein thrombosis and showed the development of collateral venous channels proximal to the ligated popliteal vein. Therefore, the principal educational value of this case lies in the decision-making process that led to ligation without venous reconstruction when conventional reconstruction was considered technically unfavorable, as well as in the recurrence of PE after a prolonged delay in definitive treatment.

### Limitations

This report has several limitations. First, this is a single case, and the findings cannot be generalized to all patients with PVA. Second, although the patient remained free of clinically apparent lower-leg edema and venous duplex ultrasonography at 1 year demonstrated no deep vein thrombosis and showed the development of collateral venous channels, longer follow-up is required to assess the risk of chronic venous insufficiency after popliteal vein ligation. Third, objective assessments of venous function, such as calf circumference, the Venous Clinical Severity Score, and air plethysmography, were not performed. In addition, although preoperative ultrasonography confirmed the patency of the great saphenous and profunda femoris veins, venous reflux and the functional adequacy of collateral venous drainage were not directly assessed. Therefore, ligation without venous reconstruction should not be routinely applied and should be considered only in carefully selected patients when reconstruction is technically unfavorable.

## CONCLUSIONS

This case highlights the importance of definitive treatment and continued follow-up in patients with PVA associated with PE. When venous reconstruction is technically challenging, ligation without reconstruction may represent a feasible alternative in carefully selected patients with adequate alternative venous drainage. Long-term surveillance remains necessary to assess the development of chronic venous insufficiency.

## SUPPLEMENTARY MATERIALS

Video 1 Intraoperative findings showing aneurysmotomy, evacuation of mobile thrombi, and ligation of the outflow segment.

## DECLARATIONS

### Availability of data and materials

The data supporting the findings of this case are available from the corresponding author upon reasonable request, while preserving patient confidentiality.

## References

[1] Bergqvist D, Björck M, Ljungman C. Popliteal venous aneurysm: a systematic review. World J Surg 2006; 30: 273–9. 10.1007/s00268-005-7982-y 16479328

[2] Maleti O, Lugli M, Collura M. Popliteal vein aneurysm : personal experience. Phlebologie 1997; 50: 53–9. (in French)

[3] Keller K, Beule J, Balzer JO, et al. Syncope and collapse in acute pulmonary embolism. Am J Emerg Med 2016; 34: 1251–7. 10.1016/j.ajem.2016.03.061 27107684

[4] Thames MD, Alpert JS, Dalen JE. Syncope in patients with pulmonary embolism. JAMA 1977; 238: 2509–11. 578884

[5] Sandstrom A, Reynolds A, Jha P. Popliteal vein aneurysm: a rare cause of pulmonary emboli. Ann Vasc Surg 2017; 38: 315.e15–7. 10.1016/j.avsg.2016.05.093 27522977

[6] Patel R, Woo K, Wakefield TW, et al. Contemporary management and outcomes of peripheral venous aneurysms: a multi-institutional study. J Vasc Surg Venous Lymphat Disord 2022; 10: 1352–8. 10.1016/j.jvsv.2022.06.011 PMC10226820 35940449

[7] Maldonado-Fernandez N, Lopez-Espada C, Martinez-Gamez FJ, et al. Popliteal venous aneurysms: results of surgical treatment. Ann Vasc Surg 2013; 27: 501–9. 10.1016/j.avsg.2012.07.005 23522443

[8] Homans J. Thrombosis of the deep veins of the lower leg, causing pulmonary embolism. N Engl J Med 1934; 211: 993–7.

[9] Donaldson GA, Linton RR, Rodkey GV. A twenty-year survey of thromboembolism at the Massachusetts General Hospital, 1939–1959. N Engl J Med 1961; 265: 208–14.

[10] Masuda EM, Kistner RL, Ferris EB 3rd. Long-term effects of superficial femoral vein ligation: thirteen-year follow-up. J Vasc Surg 1992; 16: 741–9. 1433662

[11] Emmerich J, Blachier V, Fanon L, et al. Treatment of popliteal venous aneurysms by femoral vein ligation. J Vasc Surg Cases Innov Tech 2019; 5: 228–31. 10.1016/j.jvscit.2018.11.009 PMC6598725 31297472

[12] Byerly S, Cheng V, Plotkin A, et al. Impact of ligation versus repair of isolated popliteal vein injuries on in-hospital outcomes in trauma patients. J Vasc Surg Venous Lymphat Disord 2020; 8: 437–44. 10.1016/j.jvsv.2019.09.014 31843477

[13] Zadeh RH, Farshid A, Soltani B, et al. Impact of ligation versus repair of isolated popliteal vein injuries on deep vein thrombosis and pulmonary embolism incidence in trauma patients: a meta-analysis. Langenbecks Arch Surg 2024; 409: 252. 10.1007/s00423-024-03407-8 39145795

[14] Noppeney T, Kopp R, Pfister K, et al. Treatment of popliteal vein aneurysms. J Vasc Surg Venous Lymphat Disord 2019; 7: 535–42. 10.1016/j.jvsv.2018.12.013 30853560

[15] Johnstone JK, Fleming MD, Gloviczki P, et al. Surgical treatment of popliteal venous aneurysms. Ann Vasc Surg 2015; 29: 1084–9. 10.1016/j.avsg.2015.02.009 26009475

[16] Borghese O, Pascucci D, Peluso N, et al. Popliteal venous aneurysms: a systematic review of treatment strategies and outcomes. J Clin Med 2025; 14: 3296. 10.3390/jcm14103296 PMC12111888 40429292

[17] Kawatani Y, Tajima A, Yamasaki M, et al. Popliteal venous aneurysm and pulmonary embolism initially presenting with recurrent pre-syncope: a case report. EJVES Vasc Forum 2020; 48: 19–22. 10.1016/j.ejvsvf.2020.05.008 PMC7341352 33078164

[18] Norimatsu T, Aramoto H. Surgical repair for popliteal venous aneurysm causing severe or recurrent pulmonary thromboembolism: three case reports. Ann Vasc Dis 2015; 8: 56–8. 10.3400/avd.cr.14-00111 PMC4369571 25848436

